# Type II restriction endonuclease R.Eco29kI is a member of the GIY-YIG nuclease superfamily

**DOI:** 10.1186/1472-6807-7-48

**Published:** 2007-07-12

**Authors:** Elena M Ibryashkina, Marina V Zakharova, Vladimir B Baskunov, Ekaterina S Bogdanova, Maxim O Nagornykh, Marat M Den'mukhamedov, Bogdan S Melnik, Andrzej Kolinski, Dominik Gront, Marcin Feder, Alexander S Solonin, Janusz M Bujnicki

**Affiliations:** 1 Institute of Biochemistry and Physiology of Microorganisms, Russian Academy of Sciences, Pushchino, 142290, Russia; 2 Chemistry Department, Moscow State University, Moscow, 119992, Russia; 3 Institute of Protein Research, Russian Academy of Sciences, Pushchino, 142290, Russia; 4 Faculty of Chemistry, Warsaw University, Pasteura 1, PL-02-093 Warsaw, Poland; 5 Laboratory of Bioinformatics and Protein Engineering, International Institute of Molecular and Cell Biology in Warsaw, Trojdena 4, PL-02-109 Warsaw, Poland

## Abstract

**Background:**

The majority of experimentally determined crystal structures of Type II restriction endonucleases (REases) exhibit a common PD-(D/E)XK fold. Crystal structures have been also determined for single representatives of two other folds: PLD (R.BfiI) and half-pipe (R.PabI), and bioinformatics analyses supported by mutagenesis suggested that some REases belong to the HNH fold. Our previous bioinformatic analysis suggested that REase R.Eco29kI shares sequence similarities with one more unrelated nuclease superfamily, GIY-YIG, however so far no experimental data were available to support this prediction. The determination of a crystal structure of the GIY-YIG domain of homing endonuclease I-TevI provided a template for modeling of R.Eco29kI and prompted us to validate the model experimentally.

**Results:**

Using protein fold-recognition methods we generated a new alignment between R.Eco29kI and I-TevI, which suggested a reassignment of one of the putative catalytic residues. A theoretical model of R.Eco29kI was constructed to illustrate its predicted three-dimensional fold and organization of the active site, comprising amino acid residues Y49, Y76, R104, H108, E142, and N154. A series of mutants was constructed to generate amino acid substitutions of selected residues (Y49A, R104A, H108F, E142A and N154L) and the mutant proteins were examined for their ability to bind the DNA containing the Eco29kI site 5'-CCGCGG-3' and to catalyze the cleavage reaction. Experimental data reveal that residues Y49, R104, E142, H108, and N154 are important for the nuclease activity of R.Eco29kI, while H108 and N154 are also important for specific DNA binding by this enzyme.

**Conclusion:**

Substitutions of residues Y49, R104, H108, E142 and N154 predicted by the model to be a part of the active site lead to mutant proteins with strong defects in the REase activity. These results are in very good agreement with the structural model presented in this work and with our prediction that R.Eco29kI belongs to the GIY-YIG superfamily of nucleases. Our study provides the first experimental evidence for a Type IIP REase that does not belong to the PD-(D/E)XK or HNH superfamilies of nucleases, and is instead a member of the unrelated GIY-YIG superfamily.

## Background

Type II restriction endonucleases (REases) are one of the largest groups of biochemically characterized enzymes [1]. They usually recognize a short (4–8 bp) palindromic sequence of double-stranded DNA and in the presence of Mg^2+ ^catalyze the hydrolysis of phosphodiester bonds at precise positions within or close to this sequence, leaving "blunt" ends or "sticky" (5' or 3') overhangs. REases that do not fit this definition or exhibit certain structural and functional peculiarities have been classified into several subtypes [2]. To date (May 2007), crystal structures of 24 Type II REases have been solved (for the continuously updated list see the REBASE database [1]). All but one share a common catalytic domain and a weakly conserved bipartite catalytic motif PD-(D/E)XK, suggesting that they are evolutionarily related [3]. The same core was also found in a number of other nucleases, now grouped together with the afore-mentioned REases into the "PD-(D/E)XK superfamily" [4]. Nonetheless, hundreds of known REase sequences typically exhibit little sequence similarity to one another and to other proteins, and it is not necessarily obvious that the available crystal structures are representative of all REases.

Indeed, based on sequence analyses it was predicted that REases may belong not only to the PD-(D/E)XK superfamily, but also to completely unrelated superfamilies of nucleases: PLD [5], HNH [6,7], or GIY-YIG [7]. So far, biochemical and crystallographic analyses confirmed that the catalytic domain of R.BfiI is a member of the Nuc/PLD superfamily [8]. More recently, another study first predicted that R.PabI may exhibit a different three-dimensional fold [9] and later confirmed it experimentally, revealing a new fold termed 'half-pipe' [10]. Besides, biochemical analyses have supported the prediction that the catalytic domain of R.KpnI belongs to the HNH superfamily [11]. Thus, there is experimental evidence that REases may belong to four different folds/superfamilies: PD-(D/E)XK, PLD, half-pipe, and HNH. However, compelling experimental support is still missing for the prediction that REases may also belong to the GIY-YIG superfamily [12].

Our initial bioinformatics analyses [7] revealed that REase R.Eco29kI (an enzyme from the Type II restriction-modification system Eco29kI) shares sequence similarities with the catalytic domain of GIY-YIG endonucleases, such as a homing endonuclease I-TevI. At that time, no crystal structure of any GIY-YIG superfamily member was available, therefore no three-dimensional model of the R.Eco29kI structure could be obtained to guide the analysis of sequence-structure-function relationships. We have carried out a Monte-Carlo simulation of the catalytic domain of the homing endonuclease I-TevI using spatial restraints derived from the published sparse NMR data [13] to generate the first, preliminary three-dimensional model of the GIY-YIG domain [1]. Subsequent crystallographic resolution of the I-TevI catalytic domain structure confirmed that the computational prediction of the three-dimensional fold of the GIY-YIG domain was correct [14]. Subsequently, structures of other GIY-YIG superfamily members were solved, including the 3' nuclease domain of UvrC by X-ray crystallography [15] the putative nuclease domain of a bacterial Slx-1 homolog EF2693 by NMR [16], and a comparative analysis of the GIY-YIG structures [12], providing robust templates for modeling other members of the superfamily. In this work, we present a revised sequence alignment between R.Eco29kI and the experimentally determined structure of a GIY-YIG domain, the first three-dimensional model of R.Eco29kI, and experimental validation of predicted active site residues. This is the first experimental study of R.Eco29kI that demonstrates importance of particular residues for its REase activity.

## Results

### Molecular modeling of R.Eco29kI

**Figure 1:**
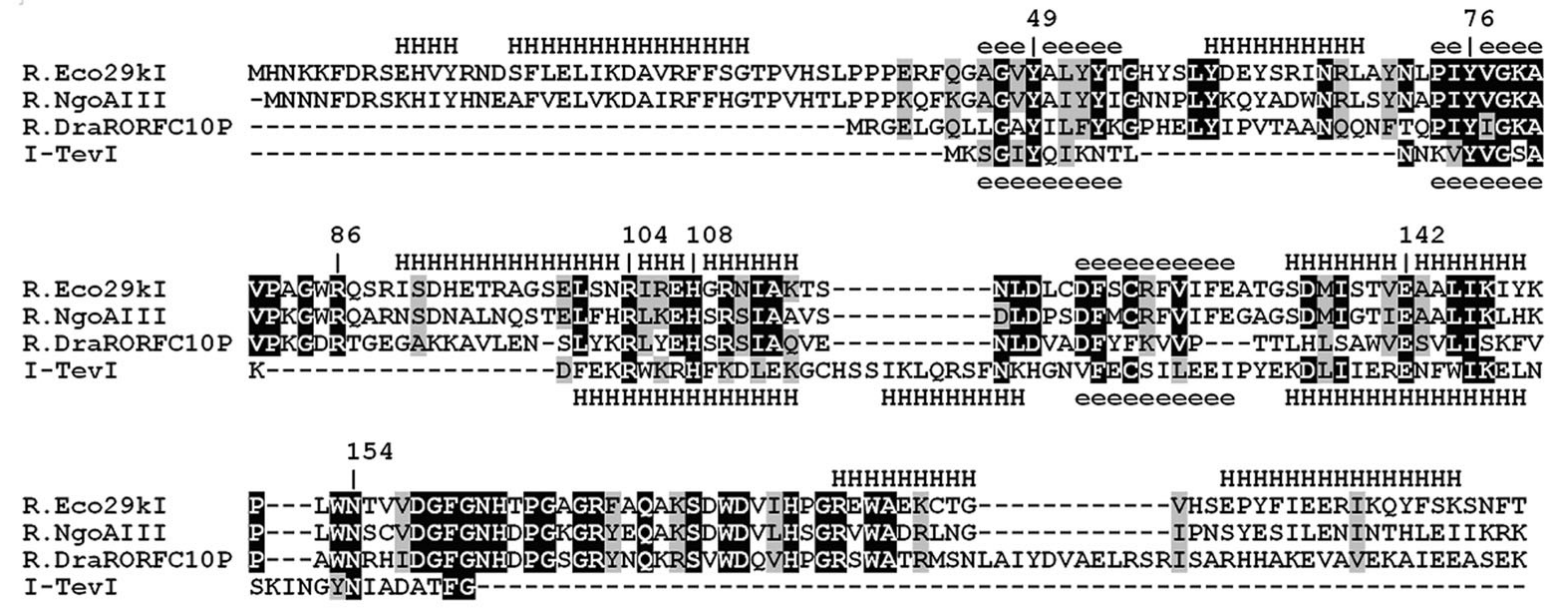
Multiple sequence alignment of R.Eco29kI and related REases with the experimentally solved structure of the catalytic domain of I-TevI homing nuclease used as a modeling template. The predicted secondary structure of R.Eco29kI is shown above the alignment, the structure of I-TevI is shown below the alignment (H, helix; e, extended). Identical residues are shown in black, the other conserved residues are in grey. Amino acid residues analyzed in this work are labeled.

The lack of overall sequence conservation among REases, the absence of invariable residues even in the active site and the presence of several alternative folds makes structure prediction and calculation of biologically relevant sequence alignments for these enzymes a non-trivial task. In order to generate an accurate three-dimensional model of R.Eco29kI structure, we used the GeneSilico meta-server to predict the secondary structure of this enzyme and to estimate the compatibility of its sequence with the structures of proteins in the Protein Data Bank using a variety of state-of-the-art fold-recognition (FR) methods (see Methods). In agreement with our earlier predictions based only on sequence similarities [7], all FR algorithms reported that R.Eco29kI sequence is compatible with the structures of GIY-YIG nucleases and not with the structures of REases from the PD-(D/E)XK superfamily currently deposited in the PDB. The consensus alignment (Figure 1) showed that the central region of the R.Eco29kI sequence (aa 44–161) can be confidently aligned to the I-TevI catalytic domain. However, the N- and C-termini did not have any counterpart in the I-TevI structure. The fold-recognition (sequence/structure) alignment in Figure 1 differs from the previously published sequence-only alignment in one very important aspect [17]: the catalytic residue R27 in I-TevI is now aligned to R104 in R.Eco29kI instead of R86.

**Figure 2:**
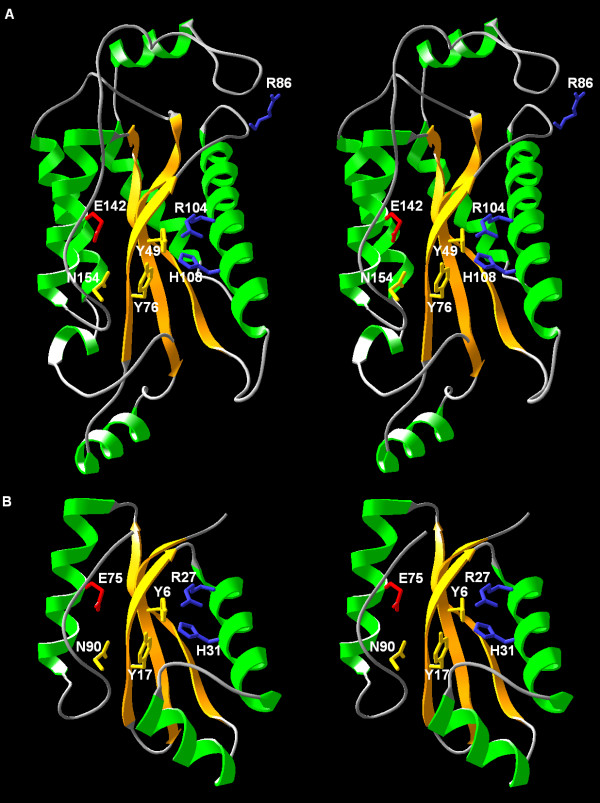
Comparison of the modeled structure of R.Eco29kI (a) and the catalytic domain of I-TevI (b). Secondary structures are colored (helices in red, strands in orange). Amino acid residues of the catalytic pocket in both enzymes and the non-essential R86 residue of R.Eco29kI are indicated and labeled. Positively charged residues are shown in blue, negatively charged residues are shown in red, neutral polar residues are shown in yellow.

The core of R.Eco29kI (aa 44–161) was homology-modeled based on the coordinates of the I-TevI structure, using the "FRankenstein's monster" protocol, which was demonstrated to be one of the most accurate methods for template-based modeling in the course of CASP5 and CASP6 experiments [18]. However, even after the optimization the predicted structure of the core was estimated to be poorly folded according to VERIFY3D (score 0.17). The long insertions with no counterparts in the template structure (aa 56–71 and 82–99 in R.Eco29kI) were generated without any restraints and hence assumed 'random coil' structures (data not shown). This is a technical limitation of all template-based modeling methods, which reflects their inability to produce 'protein-like' conformations for large polypeptide fragments that have no counterpart in the template structure. Therefore, we attempted to generate a full-length model of R.Eco29kI (including the missing termini and the poorly folded insertions) using ROSETTA and CABS – two methods employing the Monte Carlo protocol for simulation of *de novo *folding (see Materials and Methods for details). CABS was previously used to predict the I-TevI structure [17] and the topology of the *de novo *model was found to be in agreement with the crystal structure [14]. We also used the combination of FR, ROSETTA and CABS to successfully predict a number of protein structures in the recent CASP-6 experiment [19,20]. In the final full-length model of R.Eco29kI (Figure 2(a); coordinates available from [21]) all insertions and terminal extensions formed a number of regular α-helical structures, which packed quite well with one another and with the core to form an elongated globular structure of approximately 70 × 35 Å in size. Comparison with the much smaller template structure of I-TevI (Figure 2(b)) illustrates the challenge of modeling R.Eco29kI. Nonetheless, our model obtained a very good VERIFY3D score of 0.34, which suggests that it is likely to be well-folded and that potential errors are unlikely to occur in the structurally most important regions. In relation to the active site of I-TevI, the model revealed the predicted configuration of the putative active site of R.Eco29kI, comprising amino acid residues Y49, Y76, R104, H108, E142, and N154. Thus, in comparison with the previous work [17], R104 is predicted to be important for the catalytic activity of R.Eco29kI, while the model suggests that R86 is distant from the active site and therefore it is most likely not important for the REase activity.

### DNA binding properties of R.Eco29kI and mutant proteins

Mutants with amino acid substitutions (Y49A, R104A, H108F, E142A and N154L) were constructed. In accordance with the structural model of R.Eco29kI, these mutations were to affect the residues localized in the active enzyme center. We also constructed a mutant protein with an alanine substitution of R86, i.e. a residue predicted to be located in the active site in our previous analysis [17] but regarded as not important functionally, according to the current model. Using a simple purification procedure under native conditions on a Ni-NTA column, the wt and mutant proteins were isolated to 90% purity. First of all, it was necessary to ascertain that the introduced mutations did not result in perturbation of protein conformation. Ability of the mutants to specifically bind DNA containing the Eco29kI site (i.e. the 5'-CCGCGG-3' sequence recognized specifically by the R.Eco29kI REase) would demonstrate that the mutations do not perturb the overall folding of the protein, and only change the local environment of the active site.

**Figure 3:**
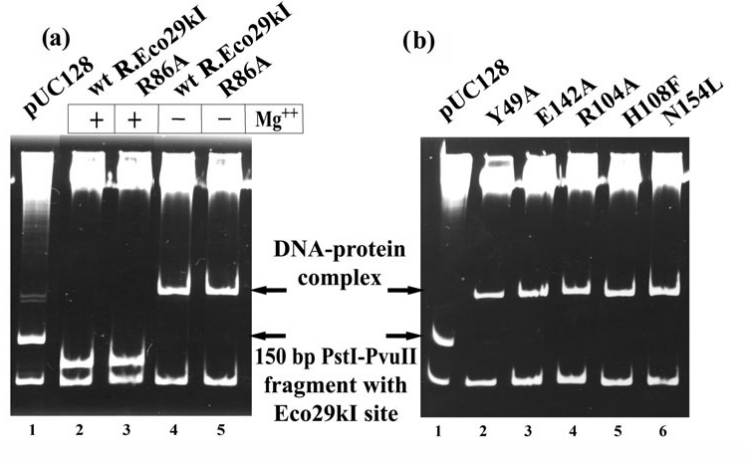
DNA binding specificity of wt R.Eco29kI and mutant proteins. (a) Effect of Mg^2+ ^ions on DNA binding by wt R.Eco29kI and R86A mutant. Lane 1, pUC128 PstI-PvuII fragments; lanes 2 and 3, reactions were carried out in the presence of Mg^2+ ^ions; lanes 4 and 5, without Mg^2+ ^ions. (b) Gel shift analysis of DNA binding by the mutant proteins in the presence of 5 mM EDTA. The names of the lanes are shown above the gel.

Type II restriction endonucleases may be divided into those that require divalent metal ions for specific binding and those forming complexes with DNA in their absence [22]. We have studied the DNA binding specificity of wt R.Eco29kI and R86A mutant by electrophoretic mobility shift assay (EMSA) with the PvuII-PstI fragments of pUC128 plasmid. The 150 bp DNA fragment contains the single R.Eco29kI site. As is shown in Figure 3(a), the binding specificity of wt R.Eco29kI and R86A is independent of the presence of Mg^2+ ^ions in the reaction mixture. Only the 150 bp DNA fragment with the recognition site was shifted by the addition of both wt R.Eco29kI and R86A (Figure 3(a), lanes 4 and 5, respectively). In the presence of Mg^2+ ^at 37°C the 150 bp fragment is cleaved by wt R.Eco29kI, as it should (Figure 3(a), lane 2). The Ala substitution of the R86 residue, which is predicted in this work to be functionally irrelevant, has no effect on the cleavage activity of the protein (Figure 3(a), lane 3). The binding specificity of all mutant proteins was studied by EMSA in the absence of Mg^2+ ^(Figure 3(b), lanes 2–6). In all cases, only the fragment containing the Eco29kI site decreased its mobility. Thus, the substitutions had no effect on the binding specificity of mutant proteins, indicating the lack of extensive conformational differences, compared to the wt R.Eco29kI.

**Figure 4:**
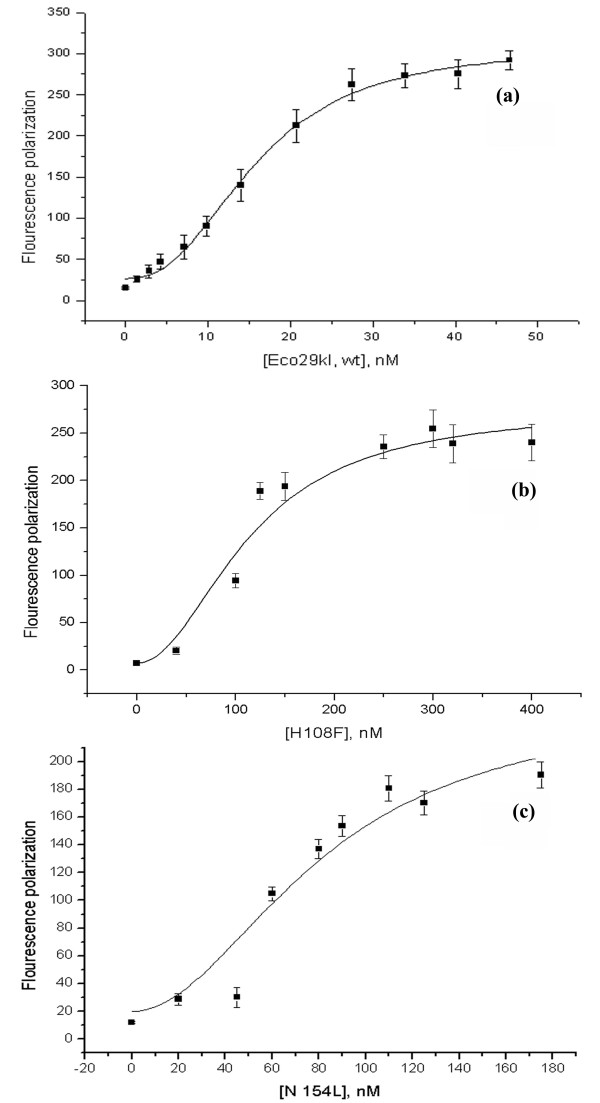
Binding of wt R.Eco29kI (a), H108F (b) and N154L (c) mutants to 24-mer DNA duplex containing the CCGCGG R.Eco29kI recognition site. The *K*_d _values were determined by direct titration using the 24-mer DNA duplex as the fluorescent probe. Serial dilutions of wild type and mutant proteins were incubated with 1 nM FAM-labeled duplex.

The ability of R.Eco29kI to specifically bind DNA in the absence of Mg^2+ ^allowed us to compare the binding efficiency of mutant proteins with the binding efficiency of wt R.Eco29kI. The fluorescence polarization measurements were made to determine the *K*_d _of wt R.Eco29kI protein and its mutant forms. For this assay, we used the 24-bp synthetic DNA duplex labeled with 5(6) carboxyfluorescein at the 5'-end. Figure 4 shows the titration of the 1 nM fluorescent labeled duplex with wt R.Eco29kI as well as with H108F and N154L mutant proteins. The binding of wt and all mutant proteins was described by sigmoidal curves, which points to a cooperative mechanism of R.Eco29kI-DNA interaction. The dissociation constants determined according to two binding models (standard bimolecular and cooperative) are presented in Table 1. The correlation coefficient R^2 ^and χ^2^-criteria indicate that the data obtained for enzyme-DNA binding analyzed by fluorescence polarization are better fit by the cooperative binding model M2 (see Methods). The magnitude of cooperativity *n *obtained for all R.Eco29kI variants was about 2 (within the experimental error) (Table 1). It is important to emphasize that the binding mechanism is described by the same model for all mutants, which evidences that the introduced mutations have no significant effect on the oligomerization state, though the binding efficiency and the values of the coefficient R^2 ^and χ^2^-criteria differ from mutant to mutant. The DNA-binding efficiency of the H108F and N154L mutants was reduced. Y49A, R104A, and E142A exhibited quite robust binding, albeit lower than in the case of wt enzyme and R86 mutant (Table 1). Although the binding efficiency of mutants changed relative to wt R.Eco29kI, the presented data on the specificity and character of binding demonstrate that the mutations have not induced dramatic perturbations of the R.Eco29kI structure.

**Table 1:** DNA cleavage and binding activities of wt R.Eco29kI and its mutants

R.Eco29kI	DNA cleavage activity [% of wt]	standard bimolecular binding model			cooperative binding model			
		*K*_d_, nM	χ^2^	R^2^	*K*_d_, nM^n^	n	χ^2^	R^2^

WT	100	16 ± 5	1500	0.78	299 ± 50	2.1 ± 0.2	360	0.95
Y49A	0	49 ± 5	105	0.97	529 ± 50	1.9 ± 0.3	31	0.99
R86A	100	21 ± 5	1232	0,82	344 ± 19	2.0 ± 0.4	415	0.95
R104A	~2	43 ± 18	442	0.94	1911 ± 356	1.8 ± 0.2	27	0.99
H108F	0	114 ± 25	1533	0.84	14285 ± 2000	2.0 ± 0.1	582	0.94
E142A	0	45 ± 11	1480	0.87	970 ± 200	1.8 ± 0.4	547	0.95
N154L	~10	50 ± 15	1267	0.75	3100 ± 700	2.0 ± 0.3	431	0.91

### DNA cleavage activity of wt R.Eco29kI and mutant proteins

**Figure 5:**
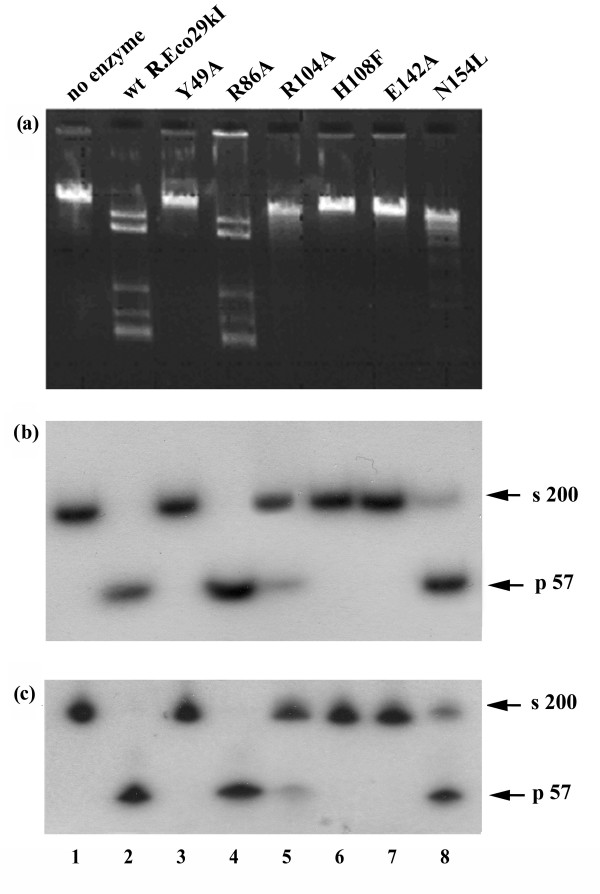
DNA cleavage activity of wt R.Eco29kI and its mutants. (a) Electrophoresis of the reaction products of phage φ80vir DNA with R.Eco29kI and mutant proteins in 0.8% agarose. (b) Electrophoresis of the cleavage products of the 200 bp DNA fragment by R.Eco29kI and mutant proteins in 5 % polyacrylamide gel under non-denaturing conditions. (c) Electrophoresis of the reaction products of the 200 bp DNA fragment with R.Eco29kI and mutant proteins in 8% polyacrylamide gel under denaturing conditions. Gel patterns with cleavage products are shown at the points after which the reaction rate ceased to rise. s – substrate, 200 bp DNA fragment with Eco29kI site; p – products, the cleavage products of 200 bp DNA fragment after treatment by R.Eco29kI.

To evaluate the catalytic activity of wt R.Eco29kI and mutant proteins we studied the cleavage of substrates with multiple recognition sites (φ80vir DNA) and with only single site (200 bp DNA fragment). The cleavage experiments revealed that mutants Y49A, H108F, and E142A exhibited no detectable activity (Figure 5(a) and 5(b), lanes 3, 6 and 7). R104A and N154L exhibited a strongly reduced activity; complete DNA cleavage was not observed (Figure 5(a) and 5(b), lanes 5 and 8). R86A is indistinguishable from wt enzyme (Figure 5(a) and 5(b), lane 4).

The activity was measured from the intensity pattern of DNA cleavage products. The activity of the mutants was calculated as percentage of the wt R.Eco29kI cleavage activity taking into account the dilution of wt enzyme and reaction incubation time. The summarized data are presented in Table 1. To test mutant proteins for specific nicking activity the reaction products of cleavage of DNA substrate with a single Eco29kI site were analyzed under denaturing conditions (Figure 5(c), lanes 2–8). Mutants Y49A, H108F, and E142A had neither double-stranded DNA (ds DNA) cleavage nor nicking activity. Comparison of the profiles of R104A and N154L hydrolysis reaction products under native and denaturing conditions demonstrates a concerted weakening of ds DNA cleavage and nicking activities.

## Discussion

Most of Type II REases characterized to date were found to belong to the PD-(D/E)XK superfamily of nucleases [23], although theoretical predictions have been made that some REases belong to different, unrelated superfamilies. To date, predictions have been experimentally confirmed for REases from three non-PD-(D/E)XK superfamilies: R.BfiI belongs to the PLD superfamily [5], R.PabI exhibits a new 'half-pipe' fold [10] and R.KpnI and a few other enzymes belong to the HNH superfamily [24]. Here, we present a refined alignment and the first structural model of the R.Eco29kI structure, and the first set of experimental data that support the prediction that REases may belong also to the GIY-YIG superfamily of nucleases.

According to the model, the active center of R.Eco29kI comprises amino acid residues Y49, Y76, R104, H108, E142, and N154. Alanine substitutions of the corresponding residues in I-TevI, the archetypal member of the GIY-YIG superfamily, have been shown to be critical for nuclease activity of the enzyme [13]. The analysis of the crystal structure of the catalytic domain I-TevI confirmed the importance of these residues for the catalytic activity of the enzyme, having demonstrated that the putative catalytic residues are located on a shallow concave surface, and E75 (E142 of R.Eco29kI) was identified as a divalent cation-binding site [14]. We constructed mutants of R.Eco29kI with amino acid substitutions Y49A, R104A, H108F, E142A and N154L, and R86A. Proteins with Ala and Phe substitutions of Y76 and H108A and N154A were not obtained. We did not find plasmids with required substitutions possibly because of toxicity of the resulting mutant proteins. We selected Phe as a replacement for His and Leu as a replacement for Asn because they are of nearly the same size and are chemically inert. The Ala substitution of R86 residue, predicted in this work to be functionally irrelevant, has no effect on the catalysis or binding as it should. The data on the specificity and mechanism of binding show that, although the introduced substitutions change the efficiency of binding of mutant protein forms, the spatial structure of constructed mutant forms of R.Eco29kI undergoes no dramatic changes.

Substitutions Y49A, H108F, and E142A completely eliminate the nuclease activity of R.Eco29kI just as I-TevI mutants Y6A and E75A. Further, substitutions R104A and N154L greatly reduce the R.Eco29kI catalytic activity, but do not abolish it completely. It is noteworthy that the residual nuclease activity was also found in the mutants of I-TevI, in which R27 and N90 (homolog's of R104 and N154 in R.EcoR29kI), were replaced by Ala [13,14]. The efficiency of DNA-binding of Y49A, R104A and E142A mutants proved to be slightly reduced, whereas in N154L and in particular in H108F it is more significantly compromised. These data show that all the above-mentioned residues are to some extent involved in DNA-binding, but in the case of Y49, R104, and E142 the effect of mutations concerns mostly catalysis, while H108 and N154 are probably mostly important for DNA binding (as of today, it is difficult to determine the contribution of these residues to catalysis, the efficiency of which obviously depends on binding). It should be emphasized that residues R27 and E75 in I-TevI, homologous to residues R104 and E142 of R.Eco29kI, are of critical importance for catalysis [14]. All these results are in very good agreement with the structural model presented in this work and with our prediction that R.Eco29kI, unlike all other REases studied to date, belongs to the GIY-YIG superfamily of nucleases [7]. Importantly, modeling combined with mutagenesis allowed us to verify and refine the previous prediction of the active site in R.Eco29kI enzyme, which was based solely on the sequence considerations [7], demonstrating the utility of the methods for protein structure prediction. We would like to emphasize that our analysis relies primarily on the detection of similarities between R.Eco29kI and I-TevI, and does not represent an attempt to identify the functionally important residues of R.Eco29kI *ab initio*. There is no direct biochemical and biophysical data on the precise role of individual residues in the catalytic mechanism of DNA cleavage by I-TevI, therefore we refrain from making excessive speculations about the exact roles of the corresponding residues in R.EcoR29kI. On the other hand, any refinement of the I-TevI mechanism of action and the studies on the function of its residues may be reflected in appropriate adjustments of the interpretation of the R.Eco29kI model presented here.

It is noteworthy that at such a low level of similarity as between R.Eco29kI and I-TevI, one can expect some local structural divergence to occur between protein structures. In particular, secondary structure elements that are not directly hydrogen-bonded are prone to mutual translations or rotations induced by repacking of side chains in the protein core. Such shifts of homologous helices have been indeed observed between the remotely related GIY-YIG nucleases I-TevI and UvrC [15]. Therefore, the root mean square deviation of the presented R.Eco29kI model from the true (currently unknown) structure of this protein can turn out to be substantial, in the range of similarity between I-TevI and UvrC, i.e. 2.9 Å for the 74 structurally superimposable core residues. Nonetheless, we are confident that the mutual orientation of major secondary structure elements that constitute the GIY-YIG fold, and the functionally important residues of R.Eco29kI studied in this work, is very accurate.

Extreme problems with the aggregation of R.Eco29kI at high concentrations (data not shown) hampered the attempts to obtain crystals or concentrated solutions suitable for NMR analyses, therefore the theoretical model of R.Eco29kI structure developed and validated in this work will serve as a starting point for computational docking and experimental analyses aimed at the identification of residues important for specific recognition of DNA by R.Eco29kI and understanding of the cleavage mechanism and architecture of the protein-DNA complex.

## Conclusion

Extreme problems with the aggregation of R.Eco29kI at high concentrations preclude the experimental characterization of its structure by X-ray crystallography or NMR. Based on bioinformatics analyses, we predicted that R.EcoR29kI is a member of the GIY-YIG superfamily of nucleases and thus, it is unrelated to all other REases studied to date. A theoretical model of the R.Eco29kI structure was constructed using a combination of the protein fold-recognition approach to detect structural templates for homology modeling and *de novo *folding of insertions and terminal extensions unique to R.Eco29kI and not present in other protein structures. Substitutions of residues Y49, R104, H108, E142 and N154 predicted by the model to be a part of the active site lead to mutant proteins with strong defects in the catalytic activity. The mutants retain the ability to bind the substrate DNA, suggesting that the effect of substitutions is limited to the active site. The experimental data support the model based on the revised alignment that identified R104 as the catalytic residue and suggested R86 not to be important. Our study provides the first experimental evidence for a Type IIP REase that does not belong to the PD-(D/E)XK or HNH superfamilies of nucleases, and is instead a member of the unrelated GIY-YIG superfamily.

## Methods

### Protein structure prediction

Secondary structure prediction and fold-recognition analysis of R.Eco29kI were carried out via the GeneSilico metaserver gateway [25]. Homology modeling of the catalytic core was carried out using the "FRankenstein's monster" approach (see [18] for a detailed description). Briefly, alternative sequence alignments between R.Eco29kI and template structures obtained from various FR servers with significant scores (all members of the GIY-YIG superfamily) were used to build preliminary models with MODELLER [26]. The preliminary models were scored by VERIFY3D [27] and the best-scoring parts from all models were merged to form a "hybrid" model.

The "hybrid" model obtained was used as a starting point for folding simulations of the complete sequence using ROSETTA [28]. The homology-modeled core of R.Eco29kI was completely "frozen" and the search of conformational space for the variable regions was restricted by the choice of fragments from known crystal structures that were compatible with the sequence and predicted secondary structure of R.Eco29kI. The final simulation of R.Eco29kI folding was conducted by the CABS method, which uses a reduced lattice representation of the protein chain [29]. Simulations in CABS were guided by the long-range spatial restraints derived from the well-scored parts of the ROSETTA models and short-range restraints derived from the predicted secondary structure in the regions absent or poorly scored in the FR model. The "decoy" models generated during the simulation were clustered using the HCPM method [30], and the most representative member of the largest cluster (in terms of the root mean square deviation, RMSD) was selected as the final model.

### Bacterial strains, plasmids and DNA

Wild-type *eco29kIR *gene was cloned in vector plasmid pQE-30 (Ap^r^) (Qiagene, USA). The DNA of pQE30 plasmid with wt *eco29kIR *gene was used as a template for PCR amplification using mutagenic oligonucleotide primers listed below (mutant variants were generated using the QuickChange Site-Directed Mutagenesis Kit by Stratagene): Y49A: 5'-GGTGCTGGGG TG**GCT**GCTCT TTACT-3'; E142A: 5'-GATTAGTACA GTT**GCG**GCCG CTCTTA-3'; R104A: 5'-CTATCTAAT**GCA**ATTAGAGA ACATGG-3'; H108F: 5'-CTAATAGAAT TAGAGAA**TTC**GGCCGAAATA TAGC-3'; N154L: 5'-GCCTTTGTGG **CTG**ACCGTTG TTGATG-3'; N154A: 5'-GCCTTTGTGG **GCT**ACCGTTG TTGATG-3'; R86A: 5'-GGTTGG**GCG**C AGTCTAGAAT TAGTG-3'; Y76A: 5'-AACCTTCCTA TT**GCT**GTTGG CAAGGC-3'; Y76F: 5'-AACCTTCCTA TT**TTC**GTTGG CAAGGC-3'. Plasmids bearing the mutant variants of *eco29kIR *gene were sequenced to make sure that the substitution of the selected codon was introduced. Strain M15 [pREP4] (Qiagene, USA)? with plasmid p29Cm (Cm^r^) bearing *eco29kIM *gene [31] was used as a host for transformation of plasmids containing the wt and mutant *eco29kIR *genes.

### Purification of wt and mutant R.Eco29kI

One-step purification of wt and mutant R.Eco29kI variants was performed under native conditions by affinity chromatography on Ni-NTA resin according to the handbook (Qiagen, USA). The protein was more than 90% homogeneous according to SDS-PAGE analysis. Protein concentration was determined by measuring OD_280 _on a Shimadzu UV-1601 spectrophotometer (Japan) with an extinction coefficient of 38120 M^-1 ^cm^-1 ^(the value was calculated using the PCGene software package).

### Electrophoretic mobility shift assay (EMSA)

We studied the DNA binding activity of wt and mutant enzymes by EMSA with the PvuII-PstI fragments of pUC128 plasmid, containing a single Eco29kI site in the 150 bp DNA fragment, in the absence of Mg^2+ ^ions. The binding reaction was performed in assay buffer AB (10 mM Tris-HCl, pH 7.5, 50 mM NaCl) with 250 nM of enzymes for 15 min at room temperature. Each sample was loaded onto 6% polyacrylamide gel. Products were analyzed under non-denaturing conditions.

### Fluorescence anisotropy measurements

All fluorescence polarization measurements were performed using the thermo-jacketed Beacon 2000 Fluorescence Polarization System. As a substrate, we used an oligonucleotide duplex with the recognition sequence of R.Eco29kI conjugated to a 5(6)-carboxyfluorescein (FAM) via a six-carbon spacer at the 5' terminus (5'-**FAM**-TTTTGGTA**CCGCGG**CCGCAAGCTT-3').

The 24 bp double-stranded oligonucleotide was prepared by annealing equimolar amounts of each strand. The DNA fragment (1 nM) was added to each diluted enzyme in AB buffer containing BSA (0.1 mg/mL) and the binding reaction mixture was incubated at room temperature for 20 min. Fluorescence polarization in each probe was measured in the static mode (measurement time 5 sec). The equilibrium binding data were analyzed using the non-linear regression according to two enzyme-DNA binding models: the standard bimolecular binding model (M1) and the cooperative binding model (M2):

E + S ↔ ES,

nE + S ↔ EnS.

In M1, the following equation was used to determine the *K*_d _values [32,33]:

mP = mP_o _+ (mP_max _- mP_0_) * (S_0 _+ E_0 _+ *K*_d _- ((S_0 _+ E_0 _+ *K*_d_)^2 ^- 4*E_0_*S_0_)^0,5^)/2*S_0_.

In the cooperative binding model M2 [34], the apparent binding constant *K*_d _app was calculated according to:

mP = mP_o _+ (mP_max _- mP_0_) *E_0_^n^*(1/*K*_d_^app^)/(1 + E_0_^n^*(1/*K*_d_^app^)),

where mP – fluorescence polarization (FP) of the DNA-enzyme complex,

mP_0 _– minimum polarization (i.e., polarization of free DNA prior to the addition of protein),

mP_max _– final polarization (i.e., polarization of DNA totally bound to protein),

S_0 _– initial concentration of the DNA duplex,

E_0 _– initial concentration of protein,

*K*_d _– dissociation constant in M1,

n – magnitude of cooperativity,

*K*_d_^app ^– apparent binding constant in M2.

*K*_d _and *K*_d_^app ^were calculated as described Baskunov *et al*[33].

### DNA cleavage assay

The double-stranded (ds) DNA cleavage activity and nicking activities of wt R.Eco29kI and the mutants were determined. The ds DNA cleavage activity was assayed using the bacteriophage φ80vir DNA [35] and 200 bp DNA fragment with the Eco29kI site. The fragment was amplified using specific primers for pUC128: M13/pUC reverse sequencing primer (-26) and M13/pUC sequencing primer (+9). Only one strand of specific DNA was 5'-labeled with [γ-^32 ^P] ATP using T4 polynucleotide kinase (Fermentas).

The reactions were carried out in 10 μl reaction buffer RB (10 mM Tris-HCl pH 7.5, 10 mM MgCl_2_, 50 mM NaCl) containing 1 μg of phage φ80vir DNA or 0.01 μM of DNA fragment. Mutant proteins and serially diluted wild type were incubated with DNA substrate at 37°C. The aliquots were removed after fixed time intervals until the reaction rate plateaued. The cleavage reaction products of φ80vir DNA were analyzed by 0.8% agarose gel electrophoresis. The cleavage reaction products of 200 bp DNA fragment were applied onto 5% polyacrylamide gel under non-denaturing conditions. Then the gel was autoradiographed.

The deoxyribonuclease activity was measured by quantifying the intensity of bands corresponding to DNA cleavage products in the PAGE analysis. Activity of mutants was calculated as percentage of the wt R.Eco29kI cleavage activity taking into account the dilution of wt enzyme and reaction incubation time [36]. The DNA nicking activity of the proteins was checked by hydrolysis of the same 200 bp DNA fragment with the Eco29kI site. In this case, aliquots were applied onto 8% polyacrylamide gel containing 7M urea. After electrophoresis, the gel was soaked in 10% acetic acid, then was dried and autoradiographed.

## Authors' contributions

JMB initiated this project, carried out the sequence alignment and created the model of the three-dimensional structure of R.Eco29kI together with collaborators (MF, DG, and AK). EMI and MVZ carried out the molecular genetic studies, participated in analysis of DNA-protein interactions and drafted the manuscript. MVZ collected the data and created the analysis procedures. MON and MMD contributed in design approaches of the protein purification. BSM and VBB analyzed the substrate-enzyme interactions and enzyme specificity. VBB performed the statistical analysis. ESB helped to perform the mutagenesis experiments. ASS conceived of the study, and participated in its design and coordination. All authors read and approved the final manuscript.

## References

[B1] Pingoud AM (2004). Restriction endonucleases.

[B2] Roberts RJ, Vincze T, Posfai J, Macelis D (2003). REBASE: restriction enzymes and methyltransferases. Nucleic Acids Res.

[B3] Pingoud A, Fuxreiter M, Pingoud V, Wende W (2005). Type II restriction endonucleases: structure and mechanism. Cell Mol Life Sci.

[B4] Kosinski J, Feder M, Bujnicki JM (2005). The PD-(D/E)XK superfamily revisited: identification of new members among proteins involved in DNA metabolism and functional predictions for domains of (hitherto) unknown function. BMC Bioinformatics.

[B5] Sapranauskas R, Sasnauskas G, Lagunavicius A, Vilkaitis G, Lubys A, Siksnys V (2000). Novel subtype of type IIs restriction enzymes. BfiI endonuclease exhibits similarities to the EDTAresistant nuclease Nuc of *Salmonella typhimurium*. J Biol Chem.

[B6] Aravind L, Makarova KS, Koonin EV (2000). SURVEY AND SUMMARY: holliday junction resolvases and related nucleases: identification of new families, phyletic distribution and evolutionary trajectories. Nucleic Acids Res.

[B7] Bujnicki JM, Radlinska M, Rychlewski L (2001). Polyphyletic evolution of type II restriction enzymes revisited: two independent sources of second-hand folds revealed. Trends Biochem Sci.

[B8] Grazulis S, Manakova E, Roessle M, Bochtler M, Tamulaitiene G, Huber R, Siksnys V (2005). Structure of the metal-independent restriction enzyme BfiI reveals fusion of a specific DNA-binding domain with a nonspecific nuclease. Proc Natl Acad Sci USA.

[B9] Ishikawa K, Watanabe M, Kuroita T, Uchiyama I, Bujnicki JM, Kawakami B, Tanokura M, Kobayashi I Discovery of a novel restriction endonuclease by genome comparison and application of a wheat-germ-based cell-free translation assay: PabI (5'GTA/C) from the hyperthermophilic archaeon Pyrococcus abyssi. Nucleic Acids Res.

[B10] Miyazono K, Watanabe M, Kosinski J, Ishikawa K, Kamo M, Sawasaki T, Nagata K, Bujnicki JM, Endo Y, Tanokura M, Kobayashi I (2007). Novel protein fold discovered in the PabI family of restriction enzymes. Nucleic Acids Res.

[B11] Saravanan M, Bujnicki JM, Cymerman IA, Rao DN, Nagaraja V (2004). Type II restriction endonuclease R.KpnI is a member of the HNH nuclease superfamily. Nucleic Acids Res.

[B12] Dunin-Horkawicz S, Feder M, Bujnicki JM Phylogenomic analysis of the GIY-YIG nuclease superfamily. BMC Genomics.

[B13] Kowalski JC, Belfort M, Stapleton MA, Holpert M, Dansereau JT, Pietrokovski S, Baxter SM, Derbyshire V (1999). Configuration of the catalytic GIY-YIG domain of intron endonuclease I-TevI: coincidence of computational and molecular findings. Nucleic Acids Res.

[B14] Van Roey P, Meehan L, Kowalski JC, Belfort M, Derbyshire V (2002). Catalytic domain structure and hypothesis for function of GIY-YIG intron endonuclease I-TevI. Nat Struct Biol.

[B15] Truglio JJ, Rhau B, Croteau DL, Wang L, Skorvaga M, Karakas E, Dellavecchia MJ, Wang H, Van Houten B, Kisker C (2005). Structural insights into the first incision reaction during nucleotide excision repair. EMBO J.

[B16] Snyder DA, Chen Y, Denissova NG, Acton T, Aramini JM, Ciano M, Karlin R, Liu J, Manor P, Rajan PA, Rossi P, Swapna GV, Xiao R, Rost B, Hunt J, Montelione GT (2005). Comparisons of NMR spectral quality and success in crystallization demonstrate that NMR and X-ray crystallography are complementary methods for small protein structure determination. J Am Chem Soc.

[B17] Bujnicki JM, Rotkiewicz P, Kolinski A, Rychlewski L (2001). Three-dimensional modeling of the I-TevI homing endonuclease catalytic domain, a GIY-YIG superfamily member, using NMR restraints and Monte Carlo dynamics. Protein Eng.

[B18] Kosinski J, Cymerman IA, Feder M, Kurowski MA, Sasin JM, Bujnicki JM (2003). A "FRankenstein's monster" approach to comparative modeling: merging the finest fragments of Fold-Recognition models and iterative model refinement aided by 3D structure evaluation. Proteins.

[B19] Kolinski A, Bujnicki JM (2005). Generalized protein structure prediction based on combination of fold-recognition with *de novo *folding and evaluation of models. Proteins.

[B20] Kosinski J, Gajda MJ, Cymerman IA, Kurowski MA, Pawlowski M, Boniecki M, Obarska A, Papaj G, Sroczynska-Obuchowicz P, Tkaczuk KL, Sniezynska P, Sasin JM, Augustyn A, Bujnicki JM, Feder M (2005). FRankenstein becomes a cyborg: the automatic recombination and realignment of Fold-Recognition models in CASP6. Proteins.

[B21] GeneSilico model repository. ftp://genesilico.pl/iamb/models/.

[B22] Pingoud A, Jeltsch A (1997). Recognition and cleavage of DNA by type-II restriction endonucleases. Eur J Biochem.

[B23] Bujnicki JM, Pingoud A (2004). Molecular phylogenetics of restriction endonucleases. Restriction Endonucleases: V 14.

[B24] Cymerman IA, Obarska A, Skowronek KJ, Lubys A, Bujnicki JM Identification of a new subfamily of HNH nucleases and experimental characterization of a representative member, HphI restriction endonuclease. Proteins.

[B25] Kurowski MA, Bujnicki JM (2003). GeneSilico protein structure prediction meta-server. Nucleic Acids Res.

[B26] Fiser A, Sali A (2003). Modeller: generation and refinement of homology-based protein structure models. Methods Enzymol.

[B27] Luthy R, Bowie JU, Eisenberg D (1992). Assessment of protein models with three-dimensional profiles. Nature.

[B28] Simons KT, Kooperberg C, Huang E, Baker D (1997). Assembly of protein tertiary structures from fragments with similar local sequences using simulated annealing and Bayesian scoring functions. J Mol Biol.

[B29] Kolinski A (2004). Protein modeling and structure prediction with a reduced representation. Acta Biochim Pol.

[B30] Gront D, Kolinski A (2005). HCPM--program for hierarchical clustering of protein models. Bioinformatics.

[B31] Zakharova MV, Beletskaya IV, Bolovin DV, Yurkova TV, Semenova LM, Solonin AS (2003). Structural plasmid evolution as a result of coupled recombinations at bom and cer sites. Mol Genet Genomics.

[B32] Reid SL, Parry D, Liu H-H, Connolly BA (2001). Binding and recognition of GATATC target sequences by the EcoRV restriction endonuclease: a study using fluorescent oligonucleotides and fluorescence polarization. Biochemistry.

[B33] Baskunov VB, Subach FV, Kolbanovskiy A, Kolbanovskiy M, Eremin SA, Johnson F, Bonala R, Geacintov NE, Gromova ES (2005). Effects of benzo[a]pyrene-deoxyguanosine lesions on DNA methylation catalyzed by EcoRII DNA methyltransferase and on DNA cleavage effected by EcoRII restriction endonuclease. Biochemistry.

[B34] Ackers GK, Johnson AD, Shea MA (1982). Quantitative model for gene regulation by lambda phage repressor. Proc Natl Acad Sci U S A.

[B35] Miller JH (1972). Experiments in Molecular Genetics.

[B36] Holtz JK, Topal MD (1994). Location of putative binding and catalytic sites of NaeI by random mutagenesis. J Biol Chem.

